# Community Perceptions of Neonatal Infection in Uganda

**DOI:** 10.1093/ofid/ofae607

**Published:** 2025-03-10

**Authors:** Phiona Nalubega, Agnes Ssali, Ritah Namugumya, Hannah G Davies, Mary Kyohere, Musa Sekikubo, Madeleine Cochet, Joseph Peacock, Philippa Musoke, Janet Seeley, Kirsty Le Doare, PROGRESS Study Author Group, Abdelmajid Djennad, Agnes Nyamaizi, Agnes Ssali, Alexander Amone, Amusa Wamawobe, Annettee Nakimuli, Caitlin Farley, Carol Nanyunja, Christine Najuka, Cleophas Komugisha, Dan R Shelley, Edward A R Portal, Ellie Duckworth, Emilie Karafillakis, Geraldine O’Hara, Godfrey Matovu, Hannah G Davies, Janet Seeley, Joseph Peacock, Juliet Nsimire, Katie Cowie, Kirsty Le Doare, Konstantinos Karampatsas, Lauren Hookham, Liberty Cantrell, Madeleine Cochet, Margaret Sewegaba, Mary Kyohere, Maxensia Owor, Melanie Etti, Merryn Voysey, Moses Musooko, Musa Sekikubo, Owen B Spiller, Patience Atuhaire, Paul T Heath, Philippa Musoke, Phiona Nalubega, Pooja Ravji, Richard Katungye, Ritah Namugumya, Rosalin Parks, Rose Azuba, Sam Kipyeko, Simon Beach, Stephen Bentley, Tim Old, Tobius Mutabazi, Valerie Tusubira, Vicki Chalker

**Affiliations:** Makerere University—Johns Hopkins University (MUJHU) Research Collaboration, Kampala, Uganda; Medical Research Council/Uganda Virus Research Institute and London School of Hygiene & Tropical Medicine Uganda Research Unit, Entebbe, Uganda; Makerere University—Johns Hopkins University (MUJHU) Research Collaboration, Kampala, Uganda; Institute for Infection and Immunity, St. George's, University of London, London, UK; Faculty of Infectious and Tropical Diseases, London School of Hygiene and Tropical Medicine, London, UK; Makerere University—Johns Hopkins University (MUJHU) Research Collaboration, Kampala, Uganda; Institute for Infection and Immunity, St. George's, University of London, London, UK; Department of Obstetrics and Gynaecology, Makerere University, Kampala, Uganda; Institute for Infection and Immunity, St. George's, University of London, London, UK; Institute for Infection and Immunity, St. George's, University of London, London, UK; Makerere University—Johns Hopkins University (MUJHU) Research Collaboration, Kampala, Uganda; Medical Research Council/Uganda Virus Research Institute and London School of Hygiene & Tropical Medicine Uganda Research Unit, Entebbe, Uganda; Department of Global Health and Development, London School of Hygiene and Tropical Medicine, London, UK; Makerere University—Johns Hopkins University (MUJHU) Research Collaboration, Kampala, Uganda; Medical Research Council/Uganda Virus Research Institute and London School of Hygiene & Tropical Medicine Uganda Research Unit, Entebbe, Uganda; Institute for Infection and Immunity, St. George's, University of London, London, UK

**Keywords:** community, cultural factors, neonatal infection, perception, qualitative research

## Abstract

**Background:**

We investigated awareness of neonatal infections among a population of pregnant women and other community members in Kampala, Uganda. We explored perceived causes of neonatal infections and perceptions of appropriate treatments.

**Methods:**

We conducted focus group discussions (FGDs) and in-depth interviews (IDIs) with 97 participants: 25 community leaders who took part in 3 FGDs, 12 pregnant women who took part in IDIs, and 60 pregnant women who took part in 8 FGDs, between November 2019 and October 2020. Data were analyzed thematically. This work formed part of the PROGRESS study, an observational cohort study undertaken in Kampala, Uganda, between November 2018 and April 2021.

**Results:**

Beliefs about causes, signs, symptoms, and treatment of infants with suspected infections impacted health-seeking behavior. Some illnesses were perceived to be caused by environmental factors while others were believed to have social or behavioral causes, such as the promiscuity of the male partner causing infections or the mother being bewitched. Local herbs and traditional remedies were the most preferred method of treatment and were commonly relied on to address various health issues rather than conventional medicines. Notably, no participant mentioned vaccines as a way of preventing infections.

**Conclusions:**

Pregnant women and community members’ understanding of the causes and treatment of neonatal illnesses were diverse, including environmental, social–behavioral, and supernatural causes, while both conventional and traditional remedies were perceived as appropriate treatments and sought accordingly. Understanding community perceptions and practices around neonatal infections is key to improving neonatal health interventions and outcomes.

Despite increasing efforts of the global community to reduce neonatal mortality [1], 2.5 million children died globally in 2018 in their first month of life, a figure that is likely to be an underestimate given the possibility of under-reporting in low- and middle-income countries [2]. Uganda has high <5-year mortality, 62.4 deaths per 1000 live births compared with the worldwide estimate of 38.4 deaths per 1000 live births, with a large proportion of deaths (27/1000 live births) occurring during the neonatal period [3, 4].

It is estimated that existing low-cost interventions, including vaccination and regular antenatal and postnatal visits to check for signs of infection, can reduce newborn deaths by up to 72% [5]. However, the scale-up of interventions in Sub-Saharan Africa has been both poor and inequitable. One of the main challenges to scaling up these interventions has been under-recognition of the behavioral and sociocultural aspects of newborn care practices, including traditional belief systems and the role of influencers in care-seeking behavior [5]. While major clinical causes of newborn morbidity and mortality are well documented, community perceptions of the causes of illnesses in newborns—as well as the impact of these on health-seeking behaviors for prevention and treatment—are not well understood in the Ugandan setting [6, 7]. In this paper, we describe pregnant women’s and community members’ awareness of neonatal infections and the perceived signs, causes, prevention methods, and treatments for neonatal infections.

This paper forms part of a supplement based on the PROGRESS study. The Progressing Group B Streptococcal Vaccines (PROGRESS) study aimed to describe the causes of infectious mortality and morbidity in pregnancy and neonates, as well as the seroepidemiology of group B streptococcal infection—the major cause of neonatal sepsis worldwide—in Kampala, Uganda [8].

## METHODS

We conducted focus group discussions (FGDs) and in-depth interviews (IDIs) between November 2019 and October 2020 to explore community leaders’ and pregnant women's perceptions of the signs, causes, prevention, and treatment of neonatal infections in urban Kampala, Uganda.

A total of 97 participants took part, including 25 community leaders who were divided into 3 FGDs, and 72 pregnant women, 12 of whom took part in IDIs and 60 of whom were divided into 8 FGDs.

### Study Setting

This study was conducted in Kawempe, an administrative division of metropolitan Kampala city. This division has the highest population of any division in Kampala district.

### Recruitment Methods

Pregnant women and community leaders were invited by the research team at Kawempe National Referral Hospital (KNRH) to take part in the study. Community leaders from the villages surrounding the hospital were identified and selected with support from the hospital Village Health Team (VHT) coordinator. We used the hospital-generated list for women who attend antenatal care at KNRH to select all women on that day across different stages of pregnancy. For the women who participated in the FGD, we grouped them into 3 groups: younger mothers (17–24 years), more mature women (>25 years), and women who had had >3 pregnancies. Community leaders were purposefully selected based on the roles they had in the community and/or in maternal vaccination activities in the community. Community leaders included religious leaders, local council leaders, village health teams, traditional birth attendants, and women leaders.

### Data Collection

Semistructured topic guides were used covering participants’ sociodemographic information, information on the participants’ knowledge of neonatal infections, and perceived signs, causes, prevention, and treatments of neonatal infections.

After providing written informed consent, participants took part in FGDs or IDIs conducted by interviewers experienced in qualitative methods, lasting between 60 and 120 minutes. For the less literate participants, friends and partners were present as witnesses during the consenting process. IDIs and some FGDs were held in private spaces at the hospital itself and within residential areas around KNRH.

English and Luganda were used during the interviews depending on the preferred language by the interviewee. One language was used in each FGD, chosen by the participant at the start of each session. Data were collected until no new themes were being covered in the data collection, suggesting that we had reached saturation.

### Data Management and Analysis

All interviews were audio-recorded and transcribed verbatim into Microsoft Word, and those conducted in Luganda were translated into the English language. The transcripts were cross-checked against the audio recordings by the research team to ensure accuracy.

A coding framework was developed based on the questions included in the topic guide and the study objectives. Anonymized transcripts were coded manually by one of the research team members using the coding framework. Data were analyzed thematically, drawing on themes based on the research objectives related to neonatal infections as well as those emerging from the data.

## RESULTS

The results were broadly categorized into 2 main themes, which were identified during analysis: (1) perceived causes of neonatal infection and (2) prevention and treatment of neonatal infections.

### Perceived Causes of Neonatal Infection

The community leaders and pregnant women perceived infections to be caused by environmental, cultural, and social–behavioral factors.

Diseases caused by different environmental conditions included malaria, meningitis, pneumonia, jaundice, colds, and cough. Some pregnant women and community leaders attributed newborn illnesses to environmental conditions such as change in temperature. For example, some pregnant women reported that pneumonia in neonates is caused by cold weather. They stated that they are usually encouraged by older people and fellow women to keep their legs close together, believing that this would prevent cold air from entering the womb, which they believe could cause pneumonia in the baby. Pregnant women and community leaders reported that when a mother acquires jaundice there is a high likelihood that her baby would also develop the condition, implying that the mother's jaundice directly affected the baby's health. Pregnant women and community leaders believed that jaundice could be caused by certain behaviors during pregnancy and postpartum, such as staying indoors for long periods of time.

Pregnant women and community leaders agreed that social–behavioral changes caused diseases such as pneumonia, jaundice, and spiritual illnesses. Some of the behaviors mentioned to have caused certain illnesses were sexual behaviors such as promiscuity of the male partner, cultural beliefs such as failure to take local herbs during pregnancy, bewitching, dietary choices such as taking concentrated juice, poor feeding or nutrition, and staying inside the house for too long.

Promiscuity was believed to cause newborn infections. Findings from the community leaders pointed to a belief that men having multiple sexual partners could lead to adverse health outcomes in newborns and pregnant women. They believed that this behavior, while prevalent in the past, had now become more common, and they attributed the increased incidence of jaundice in neonates and *amakilo* [eclampsia] during pregnancy to male partner behavior: “Men having sex with several partners would even cause an illness called amakilo [eclampsia]” (community leader).

Bewitching was perceived to be another cause of newborn infections. Participants revealed a belief that babies aged 3 months or younger can acquire a condition known as “false teeth” through bewitching practices. When the baby develops any signs of diarrhea, fever, reddish gums or signs of early teething, such signs are perceived to result from the presence of “false teeth.” Pregnant women mentioned that once a baby acquires false teeth, gums must be promptly scrubbed and teeth removed as a treatment, since they are believed to lead to the death of the baby if left in place. This traditional method of treatment involves the gouging out of unerupted teeth from the infant's mouth. They also stressed the need to act immediately once the teeth are removed: “Once they remove them [the false teeth] from the baby, they throw them on a road at a junction” (FGD pregnant woman). The practice of throwing the false teeth on a road junction was believed to impact a pregnant woman and her unborn baby positively by passing on the witchcraft to another woman; if a pregnant woman passed over the false teeth when crossing the road junction, she would herself give birth to a baby with false teeth, who would have poor health.

Dietary choices were mentioned by the participants as a serious cause of newborn health conditions. Consuming foods with a yellow color was believed to change the baby's body color to yellow. A pregnant woman during a focus group discussion said, “They [peers/community elderly women] tell us if you eat very many things which are yellow in color, for example, a concentrated juice and eating many fruits can make the baby turn yellow.” Furthermore, the participants expressed a belief that poor nutrition during pregnancy could contribute to the development of disabilities among neonates.

Social activities undertaken through goodwill by family and community members were also understood to lead inadvertently to infection. The act of allowing multiple people to carry the newly born baby could expose the child to various pathogens brought in by visitors. A community leader expressed during a focus group discussion, “Such women don’t know that a baby can easily catch infections from those people who come to carry that newly born baby.” The participants mentioned that the act of giving money to a newborn baby—a gesture of goodwill and congratulations—could inadvertently introduce unhygienic elements into the baby's environment. “Some people give those babies money not knowing that it is also unhygienic and may pass infections to babies” (community leaders’ FGD).

### Prevention and Treatment of Neonatal Illnesses

Community leaders acknowledged that clinical medicine such as antenatal care contributed to the well-being of both the mother and the baby; however, some pregnant women did not believe this. Community leaders reported that failure to attend antenatal care during pregnancy was the primary cause of newborn illnesses. However, some women held the belief that antenatal care was only necessary when they were sick, because they perceive pregnancy as a normal condition that does not require medical attention in the absence of complications. While antenatal care was not considered an essential process, they viewed it pragmatically as a means to ensure the attention of health care workers at the time of delivery:

“I just come here to get an antenatal file so that I don’t get disturbances during the time for delivery. If the health workers find out that you don’t have an antenatal file during delivery, they don’t give you much attention because you never attended antenatal with them.” (IDI pregnant woman)

The use of local herbs and traditional remedies was widely reported among the pregnant participants as a preferred method of treatment and prevention of neonatal infection. These alternative approaches were relied upon more than conventional medicine for addressing various health issues, as well as in lieu of vaccination. When discussing the prevention and treatment of neonatal illnesses, many pregnant women did not clearly differentiate infections from other diseases and symptoms of noninfective origin. For example, the consumption of certain local herbs was believed to prevent common neonatal jaundice, skin rashes, and meningitis alike. There was widespread belief among pregnant women that taking local herbs during pregnancy could have either a positive or negative impact on their health and on the health of their newborns. Pregnant women indicated that they commonly relied on the use of local herbs to maintain their health during pregnancy, and they believed that the failure to take these herbs could potentially affect women during delivery and have implications for the health of their newborn babies. Failure to take local herbs was widely believed to lead to infection in newborns. No community leader or pregnant woman mentioned vaccines as a potential preventative measure for their own health or the health of the baby.

Participants emphasized the importance of preventing stomach pain and infection for the baby when the cord was detached. These remedies involved mixing Vaseline (a term used for all forms of petroleum jelly) with mushroom or *Nakati*, a type of dark green vegetable. They explained that the Vaseline and mushroom mixture would be heated and then applied to the baby's cord. The women believed that if this prevention was neglected, the baby would experience pain and would cry continuously because the cord could become infected. One participant from a focus group discussion for women expressed this belief by stating, “When the baby's cord falls off, you have to prevent the baby from getting stomach pain. If you don’t prevent the baby, the baby will get stomach pain and he will be crying all the time.”

Community leaders and pregnant women affirmed their confidence in the use of specific herbs, such as the flowers of the *Musambya* and *Mukasa* trees (types of local herbs with yellow flowers). These were mixed, and the baby was then smeared or bathed with the mixture to treat jaundice. Community leaders emphasized the important role of traditional healers, herbalists, and older women in collecting these herbs, administering the remedies, and using them to “bath the baby to treat jaundice” (FGD community leader). Participants also mentioned relying on sunlight as a treatment for newborn illnesses like jaundice. This traditional practice involved exposing babies to the sun during specific times of the day, namely sunrise and sunset. One pregnant woman shared her personal experience and the advice she received from others regarding the treatment of jaundice in newborns, saying, “When I went back home and they told me that the baby had jaundice, they advised me to put the baby under the morning sunlight and in the evening [sunlight]” (IDI pregnant woman). Community leaders made the same recommendation: “The only and the best treatment for jaundice is putting the baby under the sun” (FGD community leader).

Pregnant women expressed a belief in a specific practice involving the use of a spoon for treatment of convulsions in babies. They explained that the appropriate course of action for treating convulsions depended on the specific health problem of the baby. They further explained that the purpose of putting a spoon in the baby's mouth during convulsions was 2-fold. First, it was aimed at preventing the baby from biting the gums, which could potentially cause injury or further complications. Second, the action of placing a spoon in the mouth was believed to allow the baby to breathe through the mouth. They assumed that convulsions could interfere with the baby's ability to breathe properly, and the placement of the spoon was thought to help alleviate this issue.

Pregnant women believed that certain illnesses in babies had a spiritual nature and required the intervention of family rituals for the baby to recover. Some women mentioned that *Kugangaara* (convulsion) could be treated using local herbs alone, while other pregnant women disagreed, saying that herbs alone were not sufficient. They instead suggested taking the baby to the father's family where special rituals are performed involving the preparation of herbs, which are put in hot water, and the baby is put in the steam for treatment, which is coupled with calling upon a traditional god (*Lubaale*) for healing. Some women also believed that prayer could lead to positive outcomes and healing for serious illnesses such as convulsions and meningitis: “I went and prayed from Pastor B's church…and my baby became fine” (pregnant women FGD).

Despite participants’ beliefs in traditional and natural treatments, pregnant women also emphasized the importance of providing immediate care and relief of pain to a newborn baby, specifically with paracetamol. They mentioned that in situations where a baby was ill and immediate medical attention or financial resources were not readily available, administering first aid at home, such as paracetamol, could help alleviate symptoms like fever before further action is taken.

## DISCUSSION

Our findings revealed that perceptions of the causes and treatment of neonatal infections included environmental, cultural, and social–behavioral factors. This can be framed using Murdock's ill-health causation theoretical model [9]. Murdock proposed that ill health is caused by multiple factors at various levels, including biological, behavioral, environmental, and socioeconomic factors. Using the model, Murdock emphasizes the importance of considering the interaction between these factors and the role of social, cultural, and political contexts in shaping health outcomes. Notably, where illnesses are perceived to be supernaturally caused, traditional medication is usually opted for, while for illnesses that are perceived to be biomedical, medical attention may be sought for treatment [9]. Consequently, understanding and respecting supernatural beliefs about illness causation held by individuals is important for clinical practitioners seeking to avoid distancing those they seek to treat.

Our study showed widespread overlap between the various causes of infection, prevention and treatment decisions, and health-seeking behavior in a large urban center in Uganda. Belief in supernatural causes of infection has been reported to have consequences for treatment-seeking behavior [10]. Indeed, some of the beliefs mentioned by our participants about the causes of “false teeth” have also been reported in other studies conducted in Uganda, where community members believed that false teeth required extraction, yet the process of extraction increases the risk of mortality [11, 12]. Concerns about “false teeth” in their children have also been found to represent an important "idiom of distress" for mothers in Eastern Uganda in response to their experience of social and political upheaval [13], highlighting the need to consider carefully how health-seeking behaviors of mothers of infants and neonates in the Ugandan context might reflect broader concerns about personal and social suffering, which may be difficult to identify based on clinical presentation alone.

The perceived causes of neonatal infections found in our study that were associated with environmental factors, social–behavioral factors, and traditional beliefs are common to other African settings, although maternal perception of several neonatal illnesses varies across different cultures and with differing socioeconomic backgrounds [14, 15]. In Ghana and Nigeria, for example, factors such as eating too many groundnuts in pregnancy, hygiene, evil spirits, or mosquito bites are believed to be the main causes of neonatal jaundice [14, 16, 17]. Interestingly, in our study, malaria in the pregnant woman or infant was not mentioned by participants, although it is endemic in Uganda.

Two studies conducted in Ethiopia reported caretakers who relied on traditional medicine for treatment of neonatal illnesses such as fever [18, 19]. Our findings are consistent with this, as our participants mentioned predominantly or exclusively using traditional medicine to treat neonates for conditions like jaundice. However, in the Nigerian context, 50% of mothers took their babies to the hospital immediately when signs of illness like jaundice were noticed [20], which may reflect important regional or cultural differences in health-seeking behavior.

Some participants, predominantly community leaders, acknowledged antenatal care as an important process that could help reduce the risk of infection to the mother and the baby. However, this contradicted the views of some women, who thought pregnancy was not a health condition that required regular antenatal care. Two studies conducted in Uganda and Kenya, respectively, showed similar findings, where pregnancy was treated as a normal condition that requires no medical attention [21, 22]. This belief could lead to a higher risk of maternal death, thus affecting progress on UN Sustainable Development Goal 3, which aims at reducing the global maternal mortality ratio to <70 per 100 000 live births [23]. However, it may also suggest new opportunities for more effective forms of public health messaging in the Ugandan context that avoid inherently medicalizing pregnancy as a life stage where not strictly necessary [24, 25].

As seen in other studies in the region, none of our participants suggested vaccination as a means of protecting themselves or infants from diseases. This has implications for maternal and infant vaccination programs and suggests a greater role for awareness raising.

As such, the results of our study provide important information for public health professionals and policy-makers about participants’ beliefs and practices around neonatal care in Uganda that can guide more sensitive and effective engagement with community groups in the future and help to inform the agenda for future research.

### Limitations

This study was conducted in an urban setting and may not be applicable to other settings such as rural areas. This study involved views of pregnant women but did not include those of their partners, who may have an influential role in pregnant women's health-seeking behaviors.

## CONCLUSIONS

The findings from this study indicate that pregnant mothers and community leaders are aware of neonatal illnesses and their signs and symptoms, but they combine both medical and spiritual/traditional understandings of the causes of those illnesses when seeking to prevent or treat infections. These findings will help focus the development of health education messages seeking to identify danger signs of babies at birth and to ensure appropriate care and treatment, including vaccines in pregnancy and infancy. Health programs need to target these beliefs more widely and integrate them with appropriate public health messaging to improve child health in Uganda going forward.

## Notes

***Acknowledgments.*** We thank the study community and respondents for their participation in this research.

The complete PROGRESS Study Author Group is comprised of Abdelmajid Djennad, Agnes Nyamaizi, Agnes Ssali, Alexander Amone, Amusa Wamawobe, Annettee Nakimuli, Caitlin Farley, Carol Nanyunja, Christine Najuka, Cleophas Komugisha, Dan R. Shelley, Edward A. R. Portal, Ellie Duckworth, Emilie Karafillakis, Geraldine O’Hara, Godfrey Matovu, Hannah G. Davies, Janet Seeley, Joseph Peacock, Juliet Nsimire, Katie Cowie, Kirsty Le Doare, Konstantinos Karampatsas, Lauren Hookham, Liberty Cantrell, Madeleine Cochet, Margaret Sewegaba, Mary Kyohere, Maxensia Owor, Melanie Etti, Merryn Voysey, Moses Musooko, Musa Sekikubo, Owen B. Spiller, Patience Atuhaire, Paul T. Heath, Philippa Musoke, Phiona Nalubega, Pooja Ravji, Richard Katungye, Ritah Namugumya, Rosalin Parks, Rose Azuba, Sam Kipyeko, Simon Beach, Stephen Bentley, Tim Old, Tobius Mutabazi, Valerie Tusubira, and Vicki Chalker.

This article was produced by PREPARE, which is part of the EDCTP2 programme supported by the European Union (grant number RIA2018V-2304-PREPARE). The views and opinions of authors expressed herein do not necessarily state or reflect those of EDCTP.

Detailed information regarding the research protocol and overall validation results for the PROGRESS study have been published separately [8].

***Author contributions.*** P.N., P.M., J.S., and K.L.D. conceived of the study. P.N., R.N., and A.S. were responsible for the methodology. P.N., R.N., A.S., M.K., M.C., J.P., and J.S. analyzed and interpreted the data. The original draft was prepared by P.N. and A.S., with further writing, review, and editing by P.N., R.N., A.S., J.P., H.G.D., M.K., P.M., M.C., M.S., K.L.D., and J.S. All authors read and approved the final version of the manuscript.

***Ethics*.** Approval for the study was granted by the Makerere University College of Health Sciences School of Medicine Research Ethics Committee (SOMREC 2018-130) and Uganda National Council for Science and Technology (UNCST HS 2496). The funders had no part in the design or analysis of this study.

***Patient consent*.** Informed consent was obtained from all subjects involved in the study.

***Financial support*.** This work was supported by the European and Developing Countries Clinical Trials Partnership (RIA2018V-2304). This work was also supported by the National Institute for Healthcare Research (KLD2019) and the Bill and Melinda Gates Foundation (OPP1189053). The funders of the study had no role in the study design, data collection, data analysis, data interpretation, writing of the manuscript, or decision to submit the manuscript for publication. The funders had no access to the data set of this study.

***Supplement sponsorship***. This article appears as part of the supplement “Neonatal and Young Infant Infection in Uganda: the PROGRESS Study,” sponsored by St George's, University of London.
